# AI-based chatbot micro-intervention for parents: Meaningful engagement, learning, and efficacy

**DOI:** 10.3389/fpsyt.2023.1080770

**Published:** 2023-01-20

**Authors:** Guido A. Entenberg, Sophie Mizrahi, Hilary Walker, Shirin Aghakhani, Karin Mostovoy, Nicole Carre, Zendrea Marshall, Gilly Dosovitsky, Daniellee Benfica, Alexandra Rousseau, Grace Lin, Eduardo L. Bunge

**Affiliations:** ^1^Department of Research, Fundación ETCI, Buenos Aires, Argentina; ^2^Children and Adolescents Psychotherapy and Technology Lab (CAPT), Palo Alto University, Palo Alto, CA, United States; ^3^Department of Psychology, International Institute for Internet Interventions i4Health, Palo Alto, CA, United States

**Keywords:** chatbot, parenting, artificial intelligence, learning, efficacy, intervention, AI

## Abstract

**Introduction:**

Mental health issues have been on the rise among children and adolescents, and digital parenting programs have shown promising outcomes. However, there is limited research on the potential efficacy of utilizing chatbots to promote parental skills. This study aimed to understand whether parents learn from a parenting chatbot micro intervention, to assess the overall efficacy of the intervention, and to explore the user characteristics of the participants, including parental busyness, assumptions about parenting, and qualitative engagement with the chatbot.

**Methods:**

A sample of 170 parents with at least one child between 2–11 years old were recruited. A randomized control trial was conducted. Participants in the experimental group accessed a 15-min intervention that taught how to utilize positive attention and praise to promote positive behaviors in their children, while the control group remained on a waiting list.

**Results:**

Results showed that participants engaged with a brief AI-based chatbot intervention and were able to learn effective praising skills. Although scores moved in the expected direction, there were no significant differences by condition in the praising knowledge reported by parents, perceived changes in disruptive behaviors, or parenting self-efficacy, from pre-intervention to 24-hour follow-up.

**Discussion:**

The results provided insight to understand how parents engaged with the chatbot and suggests that, in general, brief, self-guided, digital interventions can promote learning in parents. It is possible that a higher dose of intervention may be needed to obtain a therapeutic change in parents. Further research implications on chatbots for parenting skills are discussed.

## Introduction

Mental health issues among the child and adolescent population have been on the rise (1), and this trend has increased in recent years due to the consequences of COVID-19, such as social isolation and stress (2, 5). Problem behaviors are one of the most common disorders among children and adolescents (4–6), and parenting programs are found to be effective in reducing disruptive behaviors (7–10). However, parents usually face barriers to accessing effective treatment (11); therefore, digital mental health has become a promising and popular source of mental health support (12).

Digital parenting programs have shown promising outcomes in effectively treating behavioral problems in children and adolescents (13). Compared to face-to-face therapy, digital delivery has shown higher potential for consistency and protocol adherence, although it faces challenges in user retention (14). Since users tend to report valuing the inclusion of chat features, and interactive digital parent training programs have obtained larger effect sizes compared to non-interactive ones (15, 16), chatbots could help improve the delivery of digital parenting programs. Chatbots are computer-based programs that utilize artificial intelligence (AI) to communicate with people through text or voice and have the potential to deliver mental health interventions to a vast array of populations (i.e., adolescents and adults) (17).

Chatbots for mental health in adults have shown distinctive qualities compared to other digital interventions (18–21). However, the literature on parenting chatbots is still limited. A chatbot was designed to guide parents of newborn and preterm babies concerning stress, sleep, and nutrition (22). The chatbot reproduced human-like conversations and captured open-ended dialogs about the parents’ experiences. Overall, participants found it useful and reported having a positive experience. Parenting chatbots have also offered tools for depression screening and relaxation exercises (23). Other chatbots have focused on increasing parental involvement in their children’s education (24) and setting up interactive stories to help promote bonding between parents and their children (25).

Single-session interventions for parents have shown promising results for managing children and adolescents’ mental health problems, including anxiety (26), depression (27), and behavioral problems (28–30). These brief approaches may help to address barriers in parental engagement, such as time and scheduling constraints, geographical distance, childcare provision, and financial cost (27). However, research on parenting single-session web-based interventions for child’s behavioral problems is limited. Two previous studies on a parenting chatbot micro intervention have been reported. In the first study, Entenberg and colleagues (31) looked into the feasibility of the beta version of a parenting chatbot micro intervention designed to teach parents skills to praise their children effectively. The skills included: focus, specific, criticism-free, enthusiastic, and without delay. Out of 85 parents, 78% completed the intervention, with parents remembering an average of 3.7 out of the 5 skills. Parents reported that they were satisfied with the chatbot and that they would recommend it to other parents (7.44/10). Another study by Entenberg and colleagues (32) examined the user experience of Version 1.0 of the same micro intervention. Thus, the results were promising, parents achieved high completion rates (66.3%), and engagement (*M* = 49.8 messages), and reported high levels of satisfaction and a positive user experience. Noteworthy, micro-interventions aim to produce immediate or short-term improvements, given the brevity of the intervention, long-term gains are not expected in these studies [Bunge et al., (33, 34)]. Finding brief interventions that yield a small benefit to the user may increase the chances that they will return for continued engagement.

Research suggests that it is feasible to offer parent training through chatbots (31). However, studies on parenting chatbots are limited, and no data has been reported on chatbots’ potential efficacy in promoting parental learning and change. This study aims to describe the meaningful engagement, learning, and efficacy of the micro intervention used in the user experience study by Entenberg et al. (32). More specifically, the study analyzes the efficacy of a parenting chatbot micro intervention, the learnings attained by participants from pre to post-intervention, and the user characteristics of the parents, including their needs, their parental beliefs, and their engagement with the chatbot.

## Methods

### Participants

Participants were recruited through Facebook posts and email list advertisements. A total of 170 people participated in the study. To be included in the study, participants had to reside in Argentina and have at least one child between the age of 2–11 years old. Participants who reside outside Argentina, did not have access to a technological device and had children younger than two or older than eleven were excluded.

### Materials

#### Intervention

**FIGURE 1:**
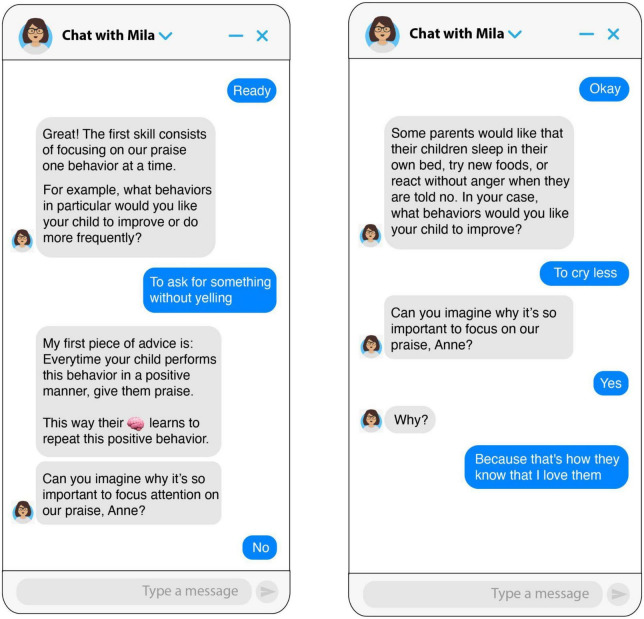
Screenshots of the chatbot teaching the focus skill.

**FIGURE 2:**
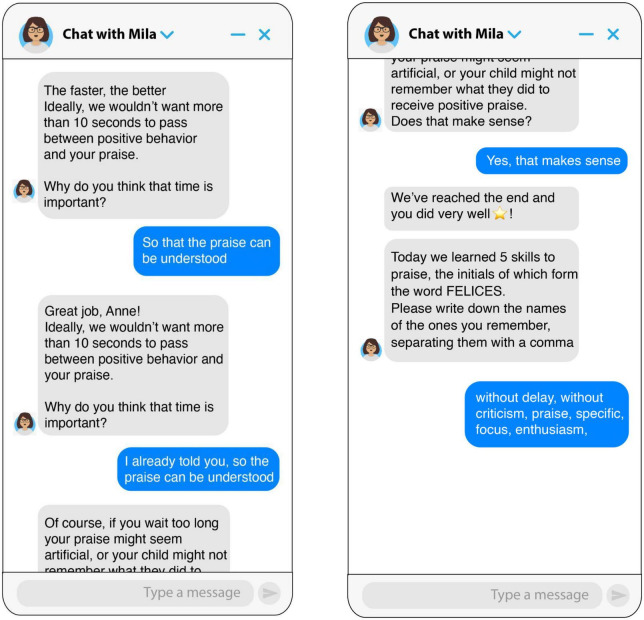
Screenshots of the chatbot teaching the without delay skill.

The parenting chatbot micro intervention was a 15-min intervention based on an initial module of the Incredible Years parenting program (10). The intervention taught parents how to utilize positive attention and praise to promote positive behaviors in their children. Five skills for effective praise were taught: focus (choose specific behavior to encourage), specific, criticism-free (avoid combining praise with criticism), enthusiasm, and without delay (praise immediately after the behavior you wish to reinforce has occurred). The intervention was designed through AI-based software developed by the company X2AI Inc. (San Francisco, CA, USA), and implemented through Facebook Messenger. This AI-based chatbot has a knowledge engine trained by researchers and psychologists to react to users’ responses and emotions reported by analyzing the content of conversations. The intervention was written in Spanish and utilized gamification and conversational design principles. Open-ended and practice questions were used to encourage user interaction throughout the intervention. Techniques such as modeling and reinforcement were also used. The skills were taught utilizing the acronym F.E.LIC.E.S., a term meaning “happy” in Spanish (see Figures 1, 2) for samples of dialog from the chatbot.

### Measures

All instruments were administered conversationally through the chatbot before, during, or at the end of the intervention.

#### Sociodemographics

A sociodemographics questionnaire was administered to each participant to obtain information about their age and gender, marital status, academic level, and employment status, as well as the age and gender of their child.

#### User characteristics

Open-ended questions were asked to learn about the participants and their experiences as parents. The first one aimed to know about parental busyness (“how busy do you think your life as a parent is?”). Answers were coded as “Very busy,” “Moderately busy,” and “Not very busy.” Prior to the skills portion of the intervention, the following two questions were presented to identify parental assumptions: (1) “What is the difference between saying” “thank you” or “thank you for listening to daddy?” (Specific skill), (2) How much time should pass between observing a good behavior and praising it? (Without delay skill). Responses were coded as correct or incorrect. Next, in order to promote interaction with the chatbot and analyze qualitative engagement, different questions were examined throughout the intervention: (1) “How do you try to make your child feel good when you spend time together?”; (2) “Which words do you use?”; (3) “Can you imagine why it is so important to focus your praise?”; and (4) “How do you show enthusiasm to your child?”

#### Learning

At the end of the intervention, an open-ended question was asked regarding which skills each participant recalled learning during the conversation. Participants typed their answers in response. The possible correct options were the five skills taught in the intervention.

#### Efficacy

To assess the efficacy of the intervention, parents were presented with the following statements and asked to rate their level of agreement using a Likert scale from 1 (“strongly disagree”) to 5 (“strongly agree”) (1) Praising knowledge: “I think I know how to praise my child when he or she deserves it.” (2) Disruptive behavior: “I often see my child misbehaving.” (3) Parenting self-efficacy: “I feel that I am well qualified to raise my child.” These statements were presented before the intervention and at 24 h follow-up.

### Procedures

**FIGURE 3:**
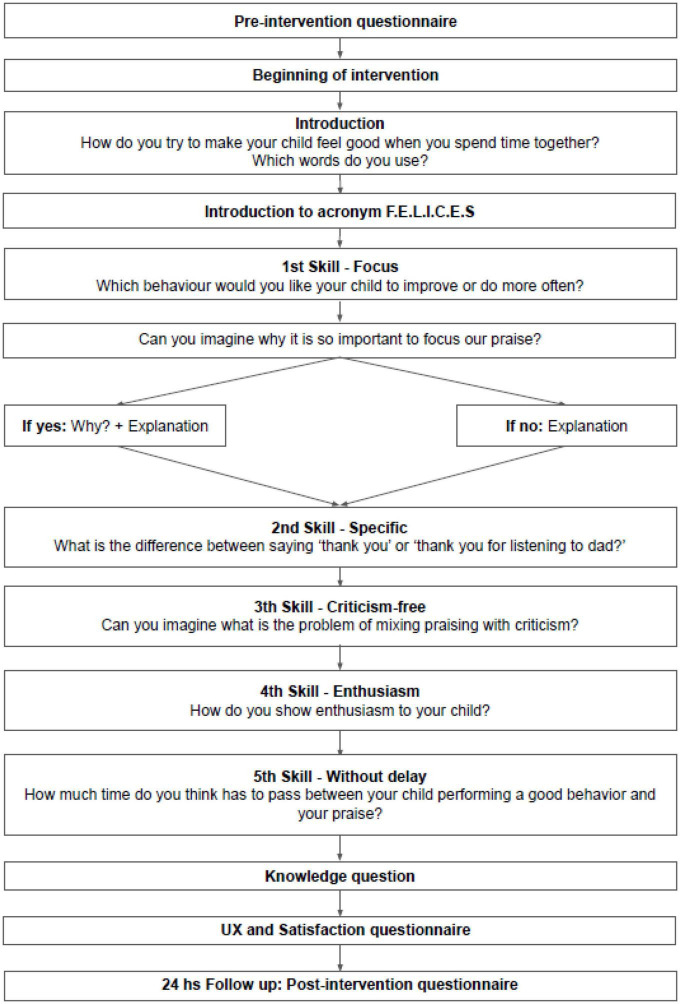
Intervention design.

A randomized control trial (RCT) was conducted. Participation was anonymous, voluntary, and unpaid. After participants were recruited, their eligibility was assessed. Those who met the inclusion criteria gave their consent electronically. All participants completed the sociodemographics and pre-intervention assessment and were randomly assigned (1:1) to the experimental or control groups. Participants in the experimental group accessed the intervention immediately, while the control group remained on a waiting list and were assessed 24 h later. After finishing the intervention, participants in the experimental group evaluated their user experience and level of satisfaction and were asked which skills they remembered. The intervention design contemplated that participants in both groups would be contacted again by the chatbot 24 h and 7 days after the end of the intervention to answer follow-up post-intervention questions (see Figure 3).

In line with ethical requirements in human research, all parents had access to the intervention after the study was completed. This study was approved by the Ethics Committee of the University of Buenos Aires, Argentina (CEI2120007).

### Data analysis

Descriptive statistics were used to analyze the responses to the questions on parental busyness and parental assumptions. Responses to qualitative engagement questions were analyzed using thematic analysis (35). Parental learning (i.e., number of skills learned) was analyzed through descriptive statistics. Mixed effects linear regressions were conducted to compare efficacy scores for the praising knowledge, disruptive behavior, and parental self-efficacy between pre-intervention and 24-h follow-up.

## Results

### Demographics

**FIGURE 4:**
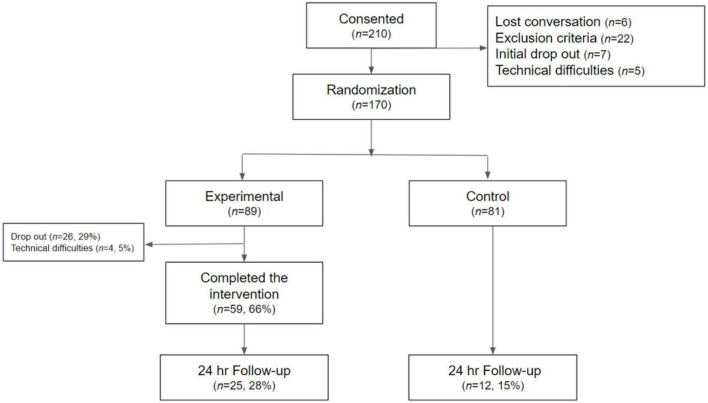
CONSORT diagram.

The total sample consisted of 170 parents; 89 were randomly assigned to the experimental group and 81 to the control group. Participants in the experimental group were mostly female (95.1%), had university/undergraduate education (73%), were married (69.7%), and most of them had a job (83.1%). The mean age of the participants’ children was 5.61 years, and their genders were evenly distributed. There were no significant differences in demographic variables between the intent-to-treat sample and the completers’ sample (see Table 1 and Figure 4) describes the CONSORT diagram.

**TABLE 1:** Sociodemographics characteristics of participants in the initial sample, depending on group assigned: experimental group or control group.

Intent to treat sample				
		Experimental *N* (89)	Control *N* (81)	*p*
Age	M (SD)	35.85 (5.77)	35.83 (7.22)	0.996
Gender	Female	77 (95.1%)	85 (95.5%)	0.586
	Male	4 (4.9%)	4 (4.5%)	
Academic level (or level of education)	Elementary	1 (1.1%)	2 (2.5%)	0.634
	High school	16 (18%)	11 (13.6%)	
	University/undergraduate	65 (73%)	64 (79%)	
	Other	7 (7.9%)	4 (4.9%)	
Marital status	Single	5 (5.6%)	4 (4.9%)	0.955
	Married	62 (69.7%)	57 (70.4%)	
	Divorced/separated	5 (5.6%)	6 (7.4%)	
	Other	17 (19.1%)	14 (17.3%)	
Working status	Employed	44 (49.4%)	38 (46.9%)	0.881
	Self-employed	30 (33.7%)	27 (33.3%)	
	Unemployed	15 (16.9%)	16 (19.8%)	
Age of child (or child’s age)	M (SD)	5.61 (2.81)	5.69 (2.93)	0.802
Gender of child (or child’s gender)	Female	44 (49.4%)	43 (53.1%)	0.635
	Male	45 (50.6%)	38 (46.9%)	

*N* = 170 (Experimental = 89; Control = 81). For group comparisons (or to compare groups) a Mann-Whitney U or a chi-square test were used, depending on the type of variable (quantitative or qualitative). *p* < 0.05.

### User characteristics

User characteristics were divided into three areas: parental busyness, parental assumptions, and qualitative engagement. A total of 28 parents responded to the question of parental busyness; 82.1% reported that they were very busy, 10.7% moderately busy, and 7.1% not very busy. In terms of parental assumptions, two questions were assessed. For the first one, 98.6% (*N* = 68) of the parents provided relevant answers and 54.4% of them correctly assumed that praise is better when it is specific. Regarding the second question (praise timing) all parents (*N* = 53) answered relevantly, and most of them correctly assumed that praise should come immediately after good conduct (96.2%).

For qualitative engagement, more than half of the parents (53.40%) said that they use attention or spending time together with their children as a way to make them feel good (e.g., “I play with him, make him laugh, or we do projects together”). For words of praise, most parents focused on performance (25.45%, “Very good! That’s how it’s done”). When asked why they think it is important to focus praise, most parents responded not knowing (36.92%, “Don’t know,” “Explain”). For ways of showing enthusiasm, the majority of parents reported using non-verbal communication without physical contact (46.57%, “With a smile, tone of voice, gestures,” “looking into the eyes and with a touch of joy”) (see Table 2).

**TABLE 2:** Qualitative engagement.

Question	Themes	Frequency (%)	Example
How do you try to make your child feel good?	Spending time together	63 (53.4%)	“We do projects together.”
	Physical contact	13 (11.02%)	“I hug him.”
	Encourage dialog/assertive communication	11 (9.32%)	“I listen to her, look her in the eye.”
	Praising for performance	10 (8.47%)	“I congratulate him.”
	Provide restraint/confidence	10 (8.47%)	“I try to make her feel safe and confident to always be herself.”
	Words of affection	7 (5.93%)	“Telling him that I love him and I am proud to be his mother.”
	Compliments for personal characteristics	3 (2.54%)	“I tell him he’s a good person.”
	Material gifts	1 (0.85%)	“With prizes, with benefits.”
Words for praise	Focus on performance	28 (25.45%)	“Very good! That’s how it’s done.”
	Focus on expression of affection	22 (20%)	“I tell my son that I love him.”
	Focus on communication/expression of ideas and feelings	15 (13.64%)	“Your opinion is very important.”
	Focus on their child’s characteristics	15 (13.64%)	“Notice what you’re saying, try to think about it, and tell me how you’re feeling.”
	Focus on time shared together	12 (10.91%)	“I love spending time with you.”
	Focus on providing support	12 (10.91%)	“I congratulate her, great job.”
	Focus on expression of pride/gratitude	6 (5.45%)	“Showing him that I like what he’s doing.”
Importance of specific praise	Don’t know	24 (36.92%)	“Explain.”
	Strengthen self-esteem and confidence	20 (30.77%)	“Because it builds confidence.”
	Identify desired behavior	18 (27.69%)	“Because we positively reinforce the behaviors we want to achieve.”
	Provide support	3 (4.62%)	“It helps them know that we are here for them.”
Ways of showing enthusiasm	Non-verbal communication 1–without physical contact (gestures, tone of voice)	34 (46.57%)	“With a smile, tone of voice; gestures.”
	Non-verbal communication 2–Physical contact (kiss, hug)	24 (32.88%)	“Hugging with a kiss”, “Looking into his eyes and hugging him.”
	Verbal communication	8 (10.96%)	“With positive words.”
	Time together	4 (5.48%)	“Accompanying them in their activities and praising their progress.”
	Material gifts	3 (4.11%)	“I gift him something he likes.”

### Learning

On average each skill was remembered by 77.96% of parents. Three skills were remembered by more than 80% of participants: criticism-free (88.1%), enthusiasm (84.7%), and without delay (83.1%) (see Table 3).

**TABLE 3:** Descriptive statistics of learned skills *N* = 59.

Variable	Label	Frequency	Percentage
Skills remembered	Focus	44	74.6%
	Specific	35	59.3%
	Criticism-free	52	88.1%
	Enthusiasm	50	84.7%
	Without delay	49	83.1%

### Efficacy

Three mixed-effects linear regressions were performed. Analyses did not include random slopes (*coefficients*) or random intercepts. There was no statistically significant effect of time (*p* = 0.51), condition (*p* = 0.30), or their interaction (*p* = 0.10) on disruptive behavior, praising knowledge (p_time_ = 0.35, p_condition_ = 0.79, p_timexcondition_ = 0.79), or parenting self-efficacy (p_time_ = 0.63, p_condition_ = 0.45, p_timexcondition_ = 0.38). Descriptive statistics for completers in the experimental group show a decrease in disruptive behavior (*M* = 0.37, *SD* = 0.96), an increase in self-efficacy (*M* = 0.21, *SD* = 0.59), and no change in the frequency of praising knowledge (see Tables 4, 5).

**TABLE 4:** Mixed-effects linear regressions.

Variable	Disruptive behaviors			Praising knowledge			Parenting self-efficacy		
	Estimator	*t*	*p*	Estimator	*t*	*p*	Estimator	*t*	*p*
Time	0.15	0.67	0.51	0.13	0.95	0.35	0.07	0.48	0.63
Condition	0.17	1.05	0.30	-0.03	-0.27	0.79	0.09	0.75	0.45
Time × condition	-0.47	-1.67	0.10	-0.05	-0.27	0.79	0.16	0.89	0.38

**TABLE 5:** Pre-24 h descriptive statistics of outcome variables.

Variable		Control group		Experimental group	
	Sample	Pre	24 h	Pre	24 h
Praising knowledge	Intent to treat	3.98	–	3.94	–
	Completers	3.67	3.92	4.16	4.16
Disruptive behavior	Intent to treat	2.89	–	3.06	–
	Completers	2.83	3	3.29	2.92
Self-efficacy	Intent to treat	3.33	–	3.43	–
	Completers	3.25	3.33	3.54	3.75

## Discussion

Mental health problems have been on the rise among children and adolescents. Digital parenting programs have shown promising results, but the potential effectiveness of using chatbots to deliver them has not yet been reported. The present study focused on assessing parents’ learning of skills and meaningful engagement through a chatbot micro intervention. The results provided insight to understand how parents engaged with the chatbot, whether they learned, and the effectiveness of the intervention.

### User characteristics

The majority of parents reported being very busy (82.1%), which has been found to be a major barrier to seeking and completing parent training (36). Furthermore, the data were collected during the first 6 months of the COVID-19 pandemic amidst increased disruptive behaviors and parental distress (37). Therefore, a brief, remote intervention may have addressed relevant issues through a flexible and convenient format.

Regarding parental assumptions, most participants knew that praise should be immediate, but nearly half didn’t know why it should be specific. Moreover, many parents (36.93%) stated that they did not know why it was important to focus on praise. Brief and written information accessible on one’s own has been reported as one of the preferred learning strategies by parents of children with mental health problems (38). Therefore, these findings show the relevance of this intervention as it provides new parenting information in an acceptable format. This is aligned with previous reports from the same participants stating that the intervention was useful for everyday life (32). Moreover, these findings can guide further considerations when developing these interventions. For example, the chatbot can briefly mention the importance of praising immediately as it reinforces previous knowledge.

Throughout the intervention parents shared relevant themes, such as how to show enthusiasm and make their children feel good. Showing attention and spending time together with their children, and conveying enthusiasm through non-verbal communication (e.g., smiling and using a gentle tone of voice) were some of the most reported strategies. Overall, these responses suggest that parents were actively engaged with the intervention and were comfortable talking to the chatbot about their relationship with their children. This is consistent with another study on chatbots for parents that reported that parents were comfortable sharing personal information and considered the chatbot trustworthy (39).

### Learning

Following the intervention, on average, each skill was remembered by 77.95% of parents. Three skills were remembered by more than 83% of the participants. This suggests that, in general, most parents had a good level of learning upon intervention. This learning rate was similar to another single-session self-guided intervention in which parenting skills were taught through a website and multimedia resources (40). Other brief interventions also enhanced parenting skills through websites, text messages, and videos (41–43). Altogether, this implies that brief, self-guided, digital interventions can promote learning in parents.

It is possible that some components of the intervention design promoted learning. Asking participants what skills they remembered after the end of the intervention may have facilitated them to recall recently acquired information and consolidate it (44). Moreover, using an acronym throughout the intervention may have acted as a memory aid (45). It is also possible that the short length of the intervention favored the learning process, as microlearning has proven useful for teaching parents brief health learning content that is limited in scope and detail (46).

### Efficacy

Participants in the experimental group reported a reduction in perceived disruptive behaviors and increased parental self-efficacy 24 h after the intervention. However, the effect of the intervention was not significant, and no significant differences were found between conditions. Thus, the interpretation of these results should be cautious. One possible explanation for this result is that parents may not have had enough time to practice the skills learned, preventing significant behavioral changes. Breitenstein and colleagues (47) have suggested that parents may need more time to absorb new information, practice learned skills, and observe changes in themselves and their children. It is also possible that the intervention dose (a single brief session) was low and that a higher dose was needed to express significant changes. Another study using text messaging was able to promote changes in parenting behaviors but a longer period of time (6 weeks) was required (48). Furthermore, it is also possible that the chatbot micro intervention would have been more effective as a treatment or prevention approach, since brief parental interventions have proven effective when targeted to at-risk or at least mildly impaired populations (30, 49, 50). Finally, in order to promote change, teaching parenting skills through a chatbot may need to be included within a more complex, general-level intervention program (39) added to face-to-face interventions.

### Limitations and future directions

One hundred and seventy participants participated in the study. The majority were female (95.1%), college-educated (73%), married (69.7%), and employed (83.1%). This suggests a homogeneous profile in the participants, and may limit the generalizability of the conclusions. Fathers’ inclusion in studies has been associated with more positive changes in children’s behavior and desirable parenting practices (51). Thus, the inclusion of more male participants could increase the effects of the intervention in further studies. Moreover, the intervention should be offered to those who need it most. Single parents, low-income, and less educated participants have been found to have higher barriers to accessing mental health diagnosis and treatment, and their children have a higher prevalence of conduct disorders (49, 52, 53).

To take part in the study, parents had to have a technological device and internet connection. This could have prevented parents with limited technological access from participating. Digital interventions have the potential to make access to interventions more affordable, but financial costs and a lack of reliable internet access can contribute to digital exclusion (54, 55). Future research could offer digital interventions in primary care settings, and individuals could be provided a technological device with internet from the facility and receive support from the staff.

Another limitation is that the efficacy measures used (i.e., praising knowledge, disruptive behavior, and parenting self-efficacy) were self-reported, and single-item Likert scales without psychometric properties were used. The rationale for using brief measures is that the assessment of the micro-intervention would be negatively affected if they included long assessments.

Specific factors may have influenced the lack of significant post-intervention changes. Some skills taught by the intervention seemed to be already known by the parents (e.g., praise without delay), therefore it is possible that they were already implementing them before this study. Furthermore, a change in the Facebook chatbots’ privacy policies during the study limited the delivery of post-intervention follow-up messages. This resulted in fewer participants completing the 24-h follow-up assessment and no participants completing it at 7 days (as was initially planned for a second follow-up in the study). This constraint may have influenced the results. Future research should examine whether parents report changes in behavior after they have had a longer period of time to practice the skills learned and observe behavioral changes.

The intervention was aimed at a wide age range (2 to 11 years). Since issues of parental concern vary across development (56–58), increasing praise skills may not motivate parents of children of certain ages to participate. The average age of the participants’ children was 5.61 years, which may have been the population that felt most attracted to learning skills to improve their relationship with their children. A further iteration of the intervention could add content on other topics (setting boundaries, communicating with pre-adolescents), to include children of a wider age range, and consider the tolls and differences these age groups experience.

## Conclusion

Mental health issues among children and adolescents are on the rise. Findings from the present study suggest that parents can receive psychoeducation and learn from a chatbot micro intervention. Parents reported being busy and had some incorrect assumptions about how to praise effectively, which shows that the intervention format was convenient and the content was relevant. Additionally, parents were able to meaningfully engage with the chatbot when communicating about their experiences with their children. While the improvements in parenting self-efficacy and the decrease in disruptive behavior were in the expected direction, they were not statistically significant, meaning that further studies with a higher intervention dose and longer follow-up times are needed. Chatbots are a unique and innovative way to increase parent training accessibility and users engage meaningfully with them. With advances in AI, chatbots for mental health are a promising intervention format for busy parents of children and adolescents.

## Data availability statement

The raw data supporting the conclusions of this article will be made available by the authors, without undue reservation.

## Ethics statement

The studies involving human participants were reviewed and approved by the Ethics Committee of the Faculty of Psychology, of the Universidad de Buenos Aires, Argentina. The patients/participants provided their written informed consent to participate in this study.

## Author contributions

GE and EB contributed to conception and design of the study and wrote the manuscript. SM organized the database, performed the statistical analysis, and wrote sections of the manuscript. HW and GD performed the statistical analysis. NC and ZM performed the qualitative analysis. SA, KM, DB, AR, and GL wrote sections of the manuscript. All authors contributed to manuscript revision, read, and approved the submitted version.

## References

[1] Olfson M Blanco C Wang S Laje G Correll C. National trends in the mental health care of children, adolescents, and adults by office-based physicians. *JAMA Psychiatry.* (2014) 71:81–90. 24285382 10.1001/jamapsychiatry.2013.3074

[2] Banerjee D Rai M. Social isolation in Covid-19: the impact of loneliness. *Int J Soc Psychiatry.* (2020) 66:525–7. 32349580 10.1177/0020764020922269 PMC7405628

[3] Nearchou F Flinn C Niland R Subramaniam S Hennessy E. Exploring the impact of COVID-19 on mental health outcomes in children and adolescents: a systematic review. *Int J Environ Res Public Health.* (2020) 17:8479. 10.3390/ijerph17228479 PMC7698263 33207689

[4] Harrison J Vannest K Davis J Reynolds C. Common problem behaviors of children and adolescents in general education classrooms in the United States. *J Emot Behav Disord.* (2012) 20:55–64. 10.4172/2471-4372.1000116 27158680 PMC4857765

[5] Kauten R Barry C. Externalizing behavior. In: Zeigler-Hill V Shackelford TK editors. *Encyclopedia of Personality and Individual Differences.* 1st ed. (Cham: Springer) (2020). p. 1509–12. 10.1007/978-3-319-24612-3

[6] McKee L Colletti C Rakow A Jones D Forehand R. Parenting and child externalizing behaviors: Are the associations specific or diffuse? *Aggress Violent Behav.* (2008) 13:201–15. 19122818 10.1016/j.avb.2008.03.005 PMC2607043

[7] Forehand R Jones D Parent J. Behavioral parenting interventions for child disruptive behaviors and anxiety: What’s different and what’s the same. *Clin Psychol Rev.* (2013) 33:133–45. 10.1016/j.cpr.2012.10.010 23178234 PMC3534895

[8] Menting A de Castro B Matthys W. Effectiveness of the Incredible Years parent training to modify disruptive and prosocial child behavior: a meta-analytic review. *Clin Psychol Rev.* (2013) 33:901–13. 10.1016/j.cpr.2013.07.006 23994367

[9] Sanders M. Triple P-positive parenting program: towards an empirically validated multilevel parenting and family support strategy for the prevention of behavior and emotional problems in children. *Clin Child Fam Psychol Rev.* (1999) 2:71–90. 10.1023/a:1021843613840 11225933

[10] Webster-Stratton C. The incredible years: parents, teachers, and children training series. *Resid Treat Child Youth.* (2001) 18:31–45.

[11] Reardon T Harvey K Baranowska M O’Brien D Smith L Creswell C. What do parents perceive are the barriers and facilitators to accessing psychological treatment for mental health problems in children and adolescents? A systematic review of qualitative and quantitative studies. *Eur Child Adolesc Psychiatry.* (2017) 26:623–47. 10.1007/s00787-016-0930-6 28054223 PMC5446558

[12] Peyton D Goods M Hiscock H. The effect of digital health interventions on parents’ mental health literacy and help seeking for their child’s mental health problem: systematic review. *J Med Internet Res.* (2022) 24:e28771. 10.2196/28771 35142623 PMC8874802

[13] Thongseiratch T Leijten P Melendez-Torres G. Online parent programs for children’s behavioral problems: a meta-analytic. *Eur Child Adolesc Psychiatry.* (2020) 29:1555–68. 10.1007/s00787-020-01472-0 31925545

[14] Breitenstein S Gross D Christophersen R. Digital delivery methods of parenting training interventions: a systematic review. *Worldviews Evid Based Nurs.* (2014) 11:168–76. 10.1111/wvn.12040 24842341

[15] Alqahtani F Orji R. Insights from user reviews to improve mental health apps. *Health Inform J.* (2020) 26:2042–66. 10.1177/1460458219896492 31920160

[16] Baumel A Pawar A Kane J Correll C. Digital parent training for children with disruptive behaviors: systematic review and meta-analysis of randomized trials. *J Child Adolesc Psychopharmacol.* (2016) 26:740–9. 10.1089/cap.2016.0048 27286325

[17] Abd-Alrazaq A Alajlani M Alalwan A Bewick B Gardner P Househ M. An overview of the features of chatbots in mental health: a scoping review. *Int J Med Inf.* (2019) 132:103978. 10.1016/j.ijmedinf.2019.103978 31622850

[18] Beatty C Malik T Meheli S Sinha C. Evaluating the therapeutic alliance with a free-text CBT conversational agent (Wysa): a mixed-methods study. *Front Digit Health.* (2022) 4:847991. 10.3389/fdgth.2022.847991 35480848 PMC9035685

[19] Dosovitsky G Bunge E. Bonding with bot: user feedback on a chatbot for social isolation. *Front Digit Health.* (2021) 3:735053. 10.3389/fdgth.2021.735053 34713203 PMC8526729

[20] Fitzpatrick K Darcy A Vierhile M. Delivering cognitive behavior therapy to young adults with symptoms of depression and anxiety using a fully automated conversational agent (Woebot): a randomized controlled trial. *JMIR Ment Health.* (2017) 4:e7785. 10.2196/mental.7785 28588005 PMC5478797

[21] Vaidyam A Wisniewski H Halamka J Kashavan M Torous J. Chatbots and conversational agents in mental health: a review of the psychiatric landscape. *Can J Psychiatry.* (2019) 64:456–64. 30897957 10.1177/0706743719828977 PMC6610568

[22] Wong J Foussat A Ting S Acerbi E Elburg R van Chien C. A Chatbot to engage parents of preterm and term infants on parental stress, parental sleep, and infant feeding: usability and feasibility study. *JMIR Pediatr Parent.* (2021) 4:e30169. 10.2196/30169 34544679 PMC8579217

[23] Chung K Cho H Park J. A chatbot for perinatal women’s and partners’ obstetric and mental health care: development and usability evaluation study. *JMIR Med Inform.* (2021) 9:e18607. 10.2196/18607 33656442 PMC7970298

[24] Wong-Villacres M Evans H Schechter D DiSalvo B Kumar N. Consejero automatico: chatbots for supporting latino parents’ educational engagement. *Proceedings of the Tenth International Conference on Information and Communication Technologies and Development.* New York, NY. (2019). p. 1–5.

[25] Zhang Z Xu Y Wang Y Yao B Ritchie D Wu T StoryBuddy: a human- AI collaborative chatbot for parent-child interactive storytelling with flexible parental involvement. *Proceedings of the CHI Conference on Human Factors in Computing Systems.* New Orleans, LA. (2022). p. 1–21.

[26] Sung J Mumper E Schleider J. Empowering anxious parents to manage child avoidance behaviors: randomized control trial of a single-session intervention for parental accommodation. *JMIR Ment Health.* (2021) 8:e29538. 10.2196/29538 34255718 PMC8292931

[27] Cardamone-Breen M Jorm A Lawrence K Rapee R Mackinnon A Yap M. A single-session, web-based parenting intervention to prevent adolescent depression and anxiety disorders: randomized controlled trial. *J Med Internet Res.* (2018) 20:e148. 10.2196/jmir.9499 29699964 PMC5945988

[28] Schleider J Weisz J. Little treatments, promising effects? Meta-analysis of single-session interventions for youth psychiatric problems. *J Am Acad Child Adolesc Psychiatry.* (2017) 56:107–15. 10.1016/j.jaac.2016.11.007 28117056

[29] Schleider J Dobias M Sung J Mullarkey M. Future directions in single-session youth mental health interventions. *J Clin Child Adolesc Psychol.* (2020) 49: 264–78. 31799863 10.1080/15374416.2019.1683852 PMC7065925

[30] Tully L Hunt C. Brief parenting interventions for children at risk of externalizing behavior problems: a systematic review. *J Child Fam Stud.* (2016) 25:705–19.

[31] Entenberg G Areas M Roussos A Maglio A Thrall J Escoredo M Using an artificial intelligence based chatbot to provide parent training: results from a feasibility study. *Soc Sci.* (2021) 10:426.

[32] Entenberg G Dosovitsky G Aghakhani S Mostovoy K Marshall Z *User Experience with a Parenting Chatbot Micro Intervention [Manuscript Submitted for Publication].* Lausanne: Frontiers in Digital Health - Human Factors and Digital Health (2022). 10.3389/fdgth.2022.989022 PMC9874295 36714612

[33] Bunge EL Williamson RE Cano M Leykin Y Muñoz RF. Mood management effects of brief unsupported internet interventions. *Internet Interv.* (2016) 5:36–43. 10.1016/j.invent.2016.06.001 30135805 PMC6096130

[34] Bunge EL Beard CL Stephens TN Leykin Y Muñoz RF. Mood management effects of a brief behavioral activation internet intervention. *J Technol Behav Sci.* (2017) 2:163–70. 10.1007/s41347-017-0026-2

[35] Braun V Clarke V. Using thematic analysis in psychology. *Qual Res Psychol.* (2006) 3:77–101.

[36] McGoron L Ondersma S. Reviewing the need for technological and other expansions of evidence-based parent training for young children. *Child Youth Serv Rev.* (2015) 59:71–83.

[37] Sullivan A Forehand R Acosta J Parent J Comer J Loiselle R COVID-19 and the acceleration of behavioral parent training telehealth: current status and future directions. *Cogn Behav Pract.* (2021) 28:618–29. 10.1016/j.cbpra.2021.06.012 34629838 PMC8488182

[38] Cunningham C Deal K Rimas H Buchanan D Gold M Sdao-Jarvie K Modeling the information preferences of parents of children with mental health problems: a discrete choice conjoint experiment. *J Abnorm Child Psychol.* (2008) 36:1123–38. 10.1007/s10802-008-9238-4 18481167

[39] Abd-Alrazaq A Alajlani M Ali N Denecke K Bewick B Househ M. Perceptions and opinions of patients about mental health chatbots: scoping review. *J Med Internet Res.* (2021) 23:e17828. 10.2196/17828 33439133 PMC7840290

[40] Korpilahti-Leino T Luntamo T Ristkari T Hinkka-Yli-Salomäki S Pulkki-Råback L Waris O Single-session, internet-based cognitive behavioral therapy to improve parenting skills to help children cope with anxiety during the COVID-19 Pandemic: Feasibility study. *J Med Internet Res.* (2022) 24:e26438. 10.2196/26438 35138265 PMC9009379

[41] Bert S Farris J Borkowski J. Parent training: implementation strategies for adventures in parenting. *J Prim Prev.* (2008) 29:243–61. 18446440 10.1007/s10935-008-0135-y

[42] Morales Chainé S Cortés Larios L Cuevas Renaud C Lira Mandujano J. Mensajes de texto en el entrenamiento a padres sobre prácticas de crianza. *Acta Investig Psicol.* (2019) 9:68–85.

[43] Scholer S Hudnut-Beumler J Dietrich M. Why parents value a brief required primary care intervention that teaches discipline strategies. *Clin Pediatr.* (2012) 51:538–45. 10.1177/0009922812439241 22496174

[44] Brown JC. A metasynthesis of the complementarity of culturally responsive and inquiry-based science education in K-12 settings: Implications for advancing equitable science teaching and learning. *J Res Sci Teach.* (2017) 54:1143–73.

[45] Azadeh N. Memory vocabulary learning strategies and long-term retention. *Int J Vocat Tech Educ.* (2009) 1:014–24.

[46] Schmidt M Glaser N Riedy T Rietta C Huszti H Wagner J Learning experience design of an mHealth intervention for parents of children with epilepsy. *Int J Med Inf.* (2022) 160:104671. 10.1016/j.ijmedinf.2021.104671 35074703 PMC9040514

[47] Breitenstein S Fogg L Ocampo E Acosta D Gross D. Parent use and efficacy of a self-administered, tablet-based parent training intervention: a randomized controlled trial. *JMIR MHealth UHealth.* (2016) 4:e5202. 10.2196/mhealth.5202 PMC4867750 27098111

[48] Downing K Salmon J Hinkley T Hnatiuk J Hesketh K. Feasibility and efficacy of a parent-focused, text message–delivered intervention to reduce sedentary behavior in 2-to 4-year-old children (Mini movers): pilot randomized controlled trial. *JMIR MHealth UHealth.* (2018) 6:e8573. 10.2196/mhealth.8573 29426816 PMC5889816

[49] Reyno S McGrath P. Predictors of parent training efficacy for child externalizing behavior problems–a meta-analytic review. *J Child Psychol Psychiatry.* (2006) 47:99–111. 10.1111/j.1469-7610.2005.01544.x 16405646

[50] Sanders M Baker S Turner KM. A randomized controlled trial evaluating the efficacy of triple P online with parents of children with early-onset conduct problems. *Behav Res Ther.* (2012) 50:675–84. 22982082 10.1016/j.brat.2012.07.004

[51] Lundahl B Tollefson D Risser H Lovejoy MC. A meta-analysis of father involvement in parent training. *Res Soc Work Pract.* (2008) 18:97–106.

[52] Ghandour R Sherman L Vladutiu C Ali M Lynch S Bitsko R Prevalence and treatment of depression, anxiety, and conduct problems in US children. *J Pediatr.* (2019) 206:256–67. 30322701 10.1016/j.jpeds.2018.09.021 PMC6673640

[53] McLanahan S Teitler J. *The Consequences of Father Absence. In: Parenting and Child Development in Nontraditional Families.* London: Psychology Press (1998). p. 91–110.

[54] Bucci S Schwannauer M Berry N. The digital revolution and its impact on mental health care. *Psychol Psychother Theory Res Pract.* (2019) 92:277–97. 10.1111/papt.12222 30924316

[55] Greer B Robotham D Simblett S Curtis H Griffiths H Wykes T. Digital exclusion among mental health service users: qualitative investigation. *J Med Internet Res.* (2019) 21:e11696. 10.2196/11696 30626564 PMC6329420

[56] Godoy L Carter A Silver R Dickstein S Seifer R. Mental health screening and consultation in primary care: The role of child age and parental concerns. *J Dev Behav Pediatr JDBP.* (2014) 35:334. 10.1097/DBP.0000000000000060 24906035 PMC4064124

[57] Howell Z Goedeke S Thorpe M. Challenges of parenting early adolescents. *Fam J.* (2021) 29:392–400. 10.1177/1066480720988273

[58] Kwon K Han S Jeon H Bingham G. Mothers’ and fathers’ parenting challenges, strategies, and resources in toddlerhood. *Early Child Dev Care.* (2013) 183:415–29. 10.1080/03004430.2012.711591

